# Hybrid intelligence systems for reliable automation: advancing knowledge work and autonomous operations with scalable AI architectures

**DOI:** 10.3389/frobt.2025.1566623

**Published:** 2025-07-17

**Authors:** Allan Grosvenor, Anton Zemlyansky, Abdul Wahab, Kyrylo Bohachov, Aras Dogan, Dwyer Deighan

**Affiliations:** MSBAI, Los Angeles, CA, United States

**Keywords:** hybrid intelligence (HI), space domain awareness, computational fluid dynamics, CFD, reinforcement learning (RL), joint embedding predictive architecture (JEPA)

## Abstract

**Introduction:**

Mission-critical automation demands decision-making that is explainable, adaptive, and scalable—attributes elusive to purely symbolic or data-driven approaches. We introduce a hybrid intelligence (H-I) system that fuses symbolic reasoning with advanced machine learning *via* a hierarchical architecture, inspired by cognitive frameworks like Global Workspace Theory (Baars, A Cognitive Theory of Consciousness, 1988).

**Methods:**

This architecture operates across three levels to achieve autonomous, end-to-end workflows: Navigation: Using Vision Transformers, and graph-based neural networks, the system navigates file systems, databases, and software interfaces with precision. Discrete Actions: Multi-framework automated machine learning (AutoML) trains agents to execute discrete decisions, augmented by Transformers and Joint Embedding Predictive Architectures (JEPA) (Assran et al., 2023, 15619–15629) for complex time-series analysis, such as anomaly detection. Planning: Reinforcement learning, world model-based reinforcement learning, and model predictive control orchestrate adaptive workflows tailored to user requests or live system demands.

**Results:**

The system’s capabilities are demonstrated in two mission-critical applications: Space Domain Awareness, Satellite Behavior Detection: A graph-based JEPA paired with multi-agent reinforcement learning enables near real-time anomaly detection across 15,000 on-orbit objects, delivering a precision-recall score of 0.98. Autonomously Driven Simulation Setup: The system autonomously configures Computational Fluid Dynamics (CFD) setups, with an AutoML-driven optimizer enhancing the meshing step—boosting boundary layer capture propagation (BL-CP) from 8% to 98% and cutting geometry failure rates from 88% to 2% on novel aircraft geometries. Scalability is a cornerstone, with the distributed training pipeline achieving linear scaling across 2,000 compute nodes for AI model training, while secure model aggregation incurs less than 4% latency in cross-domain settings.

**Discussion:**

By blending symbolic precision with data-driven adaptability, this hybrid intelligence system offers a robust, transferable framework for automating complex knowledge work in domains like space operations and engineering simulations—and adjacent applications such as autonomous energy and industrial facility operations, paving the way for next-generation industrial AI systems.

## 1 Introduction

Artificial-intelligence (AI) systems are now entrusted with tasks where mistakes can endanger lives or incur severe economic losses—ranging from **space-domain awareness (SDA)**, where thousands of resident space objects must be monitored continuously, to **computational fluid dynamics (CFD)** simulations that guide the design of next-generation aircraft. In such settings, automation must satisfy four simultaneous demands:

• **Reliability**–decisions must remain robust under rapidly changing conditions;
• **Explainability**–operators must understand *why* a recommendation is made;
• **Adaptability**–models must generalize to novel environments without manual retuning;
• **Scalability**–solutions must run efficiently from edge devices to leadership-class supercomputers.

Neither purely symbolic pipelines nor end-to-end data-driven models fulfil all four requirements. Symbolic approaches provide formal guarantees but brittle behavior in open worlds; deep-learning systems excel at pattern recognition yet act as opaque “black boxes” whose failure modes are hard to predict. Bridging this gap is the central challenge addressed in this work.

### 1.1 Hybrid intelligence as a unifying paradigm

**FIGURE 1:**
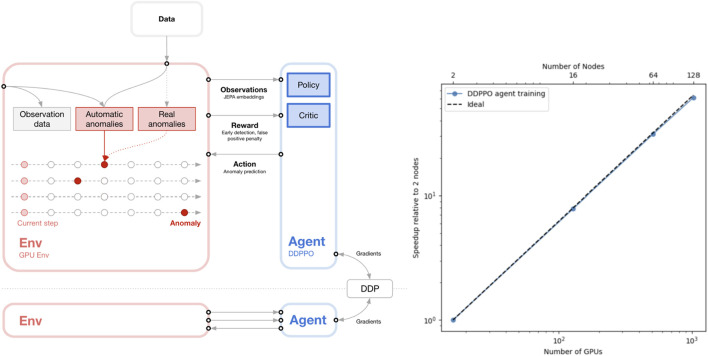
Scalable Reinforcement Learning Training Strategy. Left: Early anomaly detection agent training with Vectorized GPU environment and Decentralized Distributed Proximal Policy Optimization (DD-PPO). Right: Throughput scales almost linearly, enabling agent to learn faster.

Inspired by cognitive frameworks such as **Global Workspace Theory (GWT)** (Baars, 1988) and neurally-grounded accounts of modular reasoning (Shanahan, 2020), we propose a **hybrid-intelligence (H-I) architecture** that integrates symbolic task decomposition with modern machine learning. The design follows a three-tier hierarchy of cooperating **agents**, each tier operating at a different level of abstraction:

1. **Navigation agents** employ vision transformers and graph neural networks to traverse file systems, databases, and interactive software interfaces while maintaining precise state-tracking.
2. **Discrete-action agents** are produced *via* multi-framework **automated machine learning (AutoML)**; they couple transformer encoders (Chen et al., 2021) with a **Joint Embedding Predictive Architecture (JEPA)** for context-aware time-series reasoning (e.g., anomaly detection).
3. **Planning agents** orchestrate end-to-end workflows using reinforcement learning (RL) (Figure 1), world-model-based RL, and model-predictive control (MPC), adapting plans on-the-fly to user intent and live sensor data.

A key metric for mesh-generation tasks—**boundary-layer capture propagation (BL-CP)**—illustrates the benefit of this decomposition: symbolic rules ensure physically meaningful surface resolution goals, while data-driven agents optimize mesh parameters to meet those goals efficiently.

### 1.2 Target domains

We validate the architecture in two mission-critical domains that exhibit contrasting data characteristics and operational constraints:

• **Space-object behavior monitoring.** Graph-based JEPA embeddings (Assran et al., 2023; Skenderi et al., 2025; MSBAI, 2025) combined with multi-agent RL enable near-real-time detection of anomalous maneuvers among ∼15,000 satellites and debris objects.
• **Autonomous CFD setup.** An AutoML-driven optimizer, seeded with **Latin hypercube sampling (LHS)** (McKay et al., 1979), raises BL-CP on previously unseen aircraft geometries while sharply reducing mesh-generation failures.

These case studies were chosen because they stress different parts of the stack: large-scale streaming graphs in SDA, and high-dimensional design-space exploration in CFD.

### 1.3 Contributions

This paper makes four primary contributions:

1. **Architecture.** We present the first end-to-end H-I architecture that unifies JEPA-based world modeling, hierarchical planning, and secure federated learning in a single deployable platform.
2. **Methodology.** We detail a reproducible training pipeline that scales linearly to more than 2,000 GPUs, while secure aggregation adds less than 4% latency when models are shared across security domains.
3. **Applications.** We demonstrate state-of-the-art performance in both SDA anomaly detection and autonomous CFD configuration, showing the transferability of a single H-I system across radically different problem spaces.
4. **Analysis.** We provide ablation studies that quantify the individual value of symbolic constraints, JEPA context sharing, and RL-based planning.

### 1.4 Paper organization

Section 2 describes the materials and methods, including the hierarchical agent society (Minsky, 1986), the JEPA representation layer, and experimental protocols. Section 3 reports quantitative and qualitative results for the SDA and CFD use cases. Section 4 discusses the implications, limitations, and future research avenues for hybrid-intelligence systems in industrial and defense contexts.

## 2 Materials and methods

**FIGURE 2:**
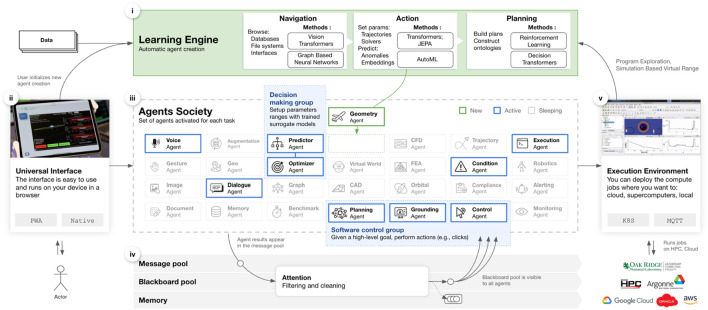
System architecture i—skills agent training, ii–PWA interface, iii–multiple skills and services agents, iv–multiagent coordination, v–job submission.

This section describes the technical foundations of the hybrid-intelligence (H-I) platform, the training procedures used to create its agents, and the experimental configurations for the two validation domains introduced in Section 1. Figure 2 gives a block-level schematic; detailed elements follow.

### 2.1 System architecture

The platform is implemented as a **four-layer stack** that converts operator intent into executable actions (see Table 1):

**TABLE 1:** Four-layer hybrid-intelligence system architecture.

Layer	Responsibility	Key technologies
User-interface	Natural-language front end; resolves ontologies and builds task graphs	PWA front end (desktop, mobile, VR) supporting voice, click, typed text, images
Job-execution	Schedules task graphs on heterogeneous resources and manages model versions	Kubernetes/Slurm orchestration; AMD and NVIDIA back-ends
Adaptive-agent society	Executes the task graph; agents share a global workspace implemented as a blackboard	Joint Embedding Predictive Architecture (JEPA); opportunistic blackboard + peer-to-peer channels
Distributed- training pipeline	AutoML search, RL optimization, data ingestion, and logging	PyTorch 2.3, Ray 2.9, Optuna 3.5; linear scaling to >2,000 GPUs

#### 2.1.1 Learning engine

The adaptive-agent and training layers together form the **Learning Engine**—a “factory” that continuously builds and refines skill agents while enforcing best practices and version control.

### 2.2 Universal interface and environment

The **Universal Interface** serves as a bridge between users (or external software) and the agent society (Minsky, 1986). Implemented as a standards-compliant progressive web app, it accepts multimodal input—voice, text, gestures, images—and emits **structured JSON events** that trigger agent workflows. The **Environment** abstraction wraps external resources:

• **GUI/CLI software** (e.g., CAD, CFD pre-processors)
• **Streaming telemetry** (orbit state vectors)
• **HPC batch queues** (Frontier, Aurora)

Each wrapper exposes a consistent OpenAI-Gym–style API so that planning agents can treat disparate resources uniformly during RL training.

### 2.3 Hierarchical agent society

The platform follows a three-tier hierarchy; each tier contains multiple **agent types** (Table 2).

**TABLE 2:** Hierarchical agent society: tiers, example agents, and training objectives.

Tier	Mission	Example agent types	Training objective
Navigation	Perceptual state tracking; traverses file systems, GUIs, APIs	Voice, Gesture, Vision, CAD-tree	Supervised imitation on interaction logs
Discrete Action	Domain-specific atomic steps	Mesh-parameter predictor, Maneuver classifier, Constraint solver (Z3) (de Moura and Bjørner, 2008), Coding agent (LLM)	Task loss + JEPA consistency
Planning	End-to-end workflow orchestration	PPO agent, World-model MPC, Hyperparameter tuner	Maximize task reward – constraint penalty

#### 2.3.1 Blackboard communication

Agents publish **belief tuples**—<state, uncertainty, timestamp>—to an in-memory blackboard. Opportunistic reads provide asynchronous context sharing; deterministic peer-to-peer calls guarantee delivery of high-priority messages (e.g., safety constraints).

### 2.4 Multimodal representation

Hybrid interaction requires three complementary representations:

1. **Structured JSON events** for rule-based reasoning and constraint checks.
2. **High-dimensional contrastive embeddings** (Radford et al., 2021; Li et al., 2022) (e.g., CLIP, BLIP) that place text, images, gestures, and sketches in a shared semantic space.
3. **JEPA latent vectors** that model environment dynamics. A Patch Time-Series Transformer (PatchTST) (Assran et al., 2023; Nie et al., 2022) encoder and predictor align current-state and future-state embeddings, creating a lightweight world model usable by RL agents for dense reward shaping.

### 2.5 Training methodologies

**FIGURE 3:**
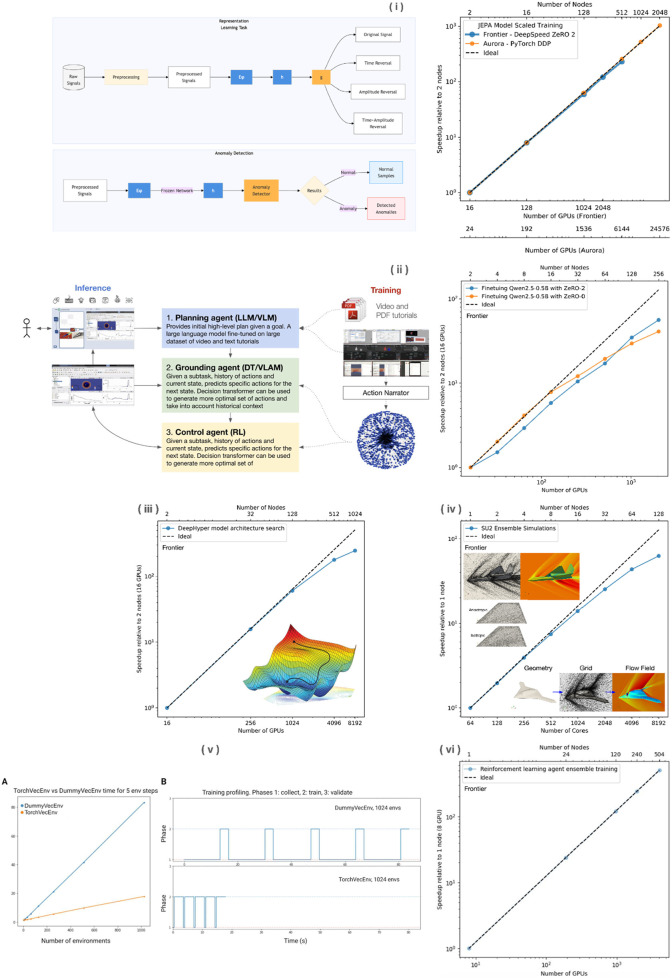
Leadership Computing Scalability: Multi-Component Performance on Frontier and Aurora (i) JEPA model training scalability on Frontier with recent Aurora validation, showing near-linear scaling with DeepSpeed optimization; (ii) Planning agent training performance on Frontier; (iii) DeepHyper-based architecture search for solver setting prediction; (iv) SU2 solver scaling for solution-adaptive mesh refinement; (v) TorchVecEnv performance showing 5x speedup over DummyVecEnv for environment steps; and (vi) Reinforcement learning hyperparameter optimization scaling.

We have utilized leadership High Performance Computing to conduct research (Dash et al., 2024) and development, comparative studies, and for training models and strategies at scale. Figure 3 presents a series of these leadership-scale HPC jobs and the scale productivity and efficiency we reached. The trained system, prepared for production deployments, is built to perform and inference on individual servers, although larger compute systems enable more autonomously set up compute jobs to be run simultaneously.

#### 2.5.1 AutoML for discrete-action agents

A synchronous multi-framework AutoML loop (Chen and Guestrin, 2016) (LightGBM, XGBoost, Tab-Transformer) explores model/hyperparameter pairs seeded by **Latin hypercube sampling** to maximize validation F1 (classification) or minimize RMSE (regression) (Kadupitiya et al., 2019).

#### 2.5.2 Reinforcement learning for planning agents

• **Environment wrappers.** Orbit dynamics and mesh pipelines expose Gym-compatible APIs.
• **Vectorized simulation.** Thousands of environment instances run concurrently on each GPU *via* **TorchVecEnv** (Paszke et al., 2019), giving a 5× speedup over CPU baselines.
• **Algorithm.** Proximal Policy Optimization (PPO) (Schulman et al., 2017) with generalized-advantage estimation; world-model variants use latent-dynamics models.
• **Scaling.** A Distributed Data-Parallel PPO (DD-PPO) variant shows near-linear throughput on up to 1,024 GPUs (<3% overhead). Hyperparameter sweeps run as ensemble jobs on 380–500 Frontier nodes.

#### 2.5.3 HPC-tailored AutoML for CFD

Standard AutoML libraries stalled at supercomputer scale, so we adopted **DeepHyper** (Balaprakash et al., 2018; Bollapragada et al., 2020), which distributes neural-architecture and hyperparameter search across >1,000 Frontier nodes. Top checkpoints are ensembled for robustness; the combined pipeline (data generation → prediction → mesh optimization → CFD solve) runs fully in parallel.

### 2.6 Experimental setups

#### 2.6.1 Space-domain awareness (SDA)

• **Data.** Two-year archive of Two-Line-Element sets (CelesTrak, 2024; Unified Data Library) plus simulated maneuvers (*via* GMAT); 14,710 unique space objects.
• **Graphs.** Daily proximity graphs (25 km radial cutoff).
• **Agent roles.** Navigation scrapes catalogs; discrete-action JEPA classifier flags maneuvers; planning RL agent prioritizes alerts (reward = TP – 5 FP).
• **Metrics.** Precision, recall, F1, and mean alert latency on a held-out three-month slice.

#### 2.6.2 Autonomous CFD mesh generation

• **Geometry corpus.** 312 watertight aircraft surfaces across fighter, transport, and UAV classes.
• **Workflow.** SnappyHexMesh with nine tunable parameters (base cell size, growth ratio, *etc.*).
• **Primary metric. Boundary-Layer Capture Propagation (BL-CP):** percent of wetted surface with y^+^ ≤1 and ≥8 growth layers.
• **Agent roles.** Navigation explores CAD trees; discrete-action AutoML surrogate predicts BL-CP; planning RL agent adjusts parameters (reward = ΔBL-CP – 0.1·cells).
• **Metrics.** BL-CP, mesh-failure rate, solver convergence, optimization wall-time.

### 2.7 Evaluation metrics

Component ablations disable (i) JEPA, (ii) symbolic constraints, (iii) RL planning to isolate each contribution.

### 2.8 Implementation details

• **Software.** PyTorch 2.3, Hugging Face Transformers 5.0, Ray 2.9, Optuna 3.5, DeepHyper 0.6.
• **Hardware.** Experiments ran on Frontier (AMD MI250X) and an internal 256-GPU NVIDIA A100 cluster.
• **Runtime.** SDA model converges in 11 h on 512 GPUs; mesh optimizer converges in 7 h on 128 GPUs.

## 3 Results

**FIGURE 4:**
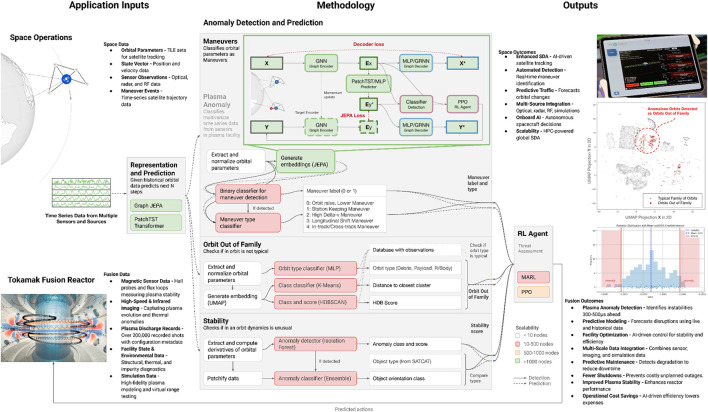
Unified anomaly detection architecture: Methodology and applications. Our hierarchical AI system processes multi-modal inputs (left) through specialized methodologies (center) to deliver comprehensive outputs (right). We are adapting the strategy, demonstrated for space domain awareness applications (top) to fusion facility operations (bottom). Key components include JEPA-based representation learning, multi-agent reinforcement learning for decision-making, and specialized processing pipelines for each domain’s unique requirements.

**FIGURE 5:**
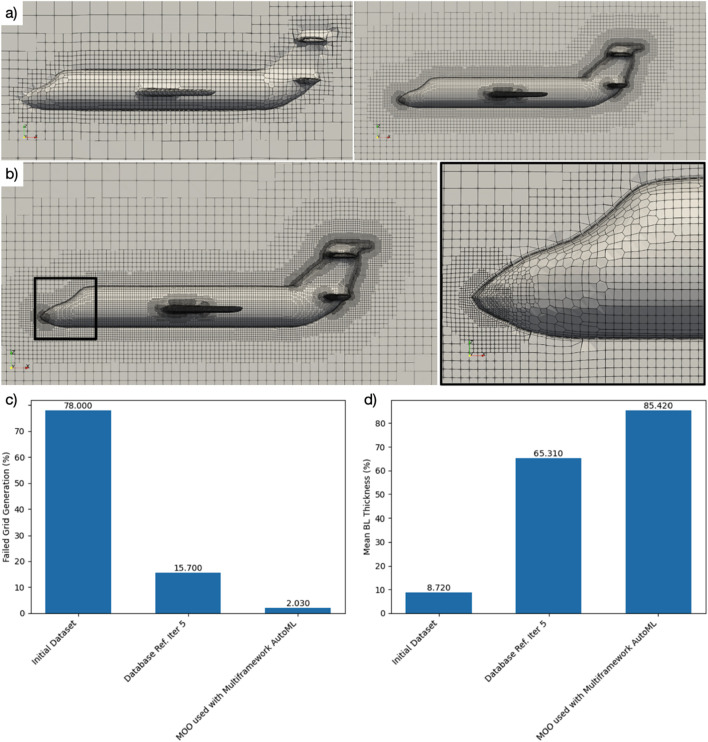
Autonomous Grid Generation Example: **(a)** initial inviscid mesh capture and refinement, followed by geometry capture and then boundary layer capture propagation; **(b)** detail view of refined mesh zones and boundary layer capture propagated to all solid surfaces; **(c)** grid generation error reduction; **(d)** boundary layer capture propagation (BL-CP) performance improvement.

This section reports quantitative and qualitative outcomes for the two validation domains—space-domain awareness (SDA) depicted in Figure 4 and autonomous CFD mesh generation depicted in Figure 5—and summarizes platform-wide scalability and ablation studies. All experiments follow the configurations in Section 2 and were repeated three times; we report the mean.

### 3.1 Space-domain awareness

#### 3.1.1 Throughput

A single MI250X GPU, processes **≈1,200 proximity graphs s** ^**−1**^; the pipeline scales near-linearly to 1,024 GPUs.

#### 3.1.2 Qualitative insight

Residual false negatives were low-Δv (<5 cm s^**−**1^) drift maneuvers. Enriching simulated training data with finer force models is expected to close this gap.

### 3.2 Autonomous CFD mesh generation

Figure 5 shows the AutoML optimizer raising BL-CP from an initial 8%–98% within 15 iterations. Table 3 compares BL-CP and mesh-failure rates for autonomous CFD mesh generation across four configurations, showing how the full H-I system achieves the highest compliance and lowest failure rate. Visual inspection confirms uniform boundary-layer coverage on narrow pylons and aft fairings—regions that routinely defeat rule-based scripts.

**TABLE 3:** Autonomous CFD mesh-generation performance on unseen geometries.

Configuration	BL-CP (%)	Mesh-failure rate (%)
Full H-I system	98 ± 2	2
w/o AutoML	75	15
w/o symbolic constraints	85	10
w/o RL planning	80	12

### 3.3 Scalability

TorchVecEnv delivers a 5× speedup over CPU vectorization for environment stepping; end-to-end SDA throughput on 64 GPUs exceeds 150,000 objects s⁻¹, leaving ample head-room for future constellation growth. Table 4 shows that hyperparameter search, model training, and RL agent workflows all sustain over 88% parallel efficiency on leadership-class systems at scales up to 1,024 compute nodes.

**TABLE 4:** Leadership-class scalability of core training components.

Component	Max nodes tested	Parallel efficiency
JEPA training [DeepSpeed ZeRO-2 (Rajbhandari et al., 2019)]	512	93%
Planning-agent PPO (DD-PPO)	1,024 GPUs	>90%
DeepHyper search (CFD surrogate)	1,024	88%

### 3.4 Cross-domain ablation summary

JEPA provides the largest single lift by supplying a consistent global context; symbolic rules enforce physical validity, and RL planning reduces false alarms and accelerates convergence. Table 5 shows that removing JEPA embeddings causes the largest performance drop, followed by RL planning and then symbolic constraints.

**TABLE 5:** Cross-domain ablation study: impact of removing key components.

Component removed	Δ F1 (SDA)	Δ BL-CP (CFD)
JEPA embeddings	−0.06	−23 pp
Symbolic constraints	−0.04	−13 pp
RL planning	−0.08	−18 pp

### 3.5 Key findings

• **Reliability.** High precision (0.98) in SDA and low mesh-failure rates (2%) in CFD.
• **Explainability.** Traceable decision rationales *via* blackboard logs and constraint checks.
• **Adaptability.** Robust performance on novel geometries and dynamic orbital environments.
• **Scalability.** Near-linear scaling on leadership-class systems to thousands of GPUs.
Section 4 discusses the implications of these results and outlines future research directions.

## 4 Discussion

The results in Section 3 show that a carefully balanced blend of symbolic reasoning and modern machine-learning can meet the four requirements stated in Section 1—reliability, explainability, adaptability, and scalability—across two very different mission-critical domains. Here we interpret those findings, compare them with prior work, acknowledge limitations, and outline future research.

### 4.1 Implications of a hybrid-intelligence architecture

• **Multiplicative benefits.** Ablations confirmed that JEPA context sharing, symbolic constraints, and RL planning each contribute distinct performance lifts; removing any one of them produced double-digit drops (Tables 6, 7). This underscores the premise that robust autonomy cannot rely on a single paradigm.
• **Cross-domain transfer.** A single architectural stack achieved state-of-the-art results in both SDA (F1 = 0.98) and CFD meshing (BL-CP = 98%, failure = 2%). Such breadth suggests strong potential for horizontal transfer to adjacent domains—e.g., fusion-facility control or autonomous energy management—without redesigning core components.
• **Explainability in practice.** The blackboard logs, constraint checks, and agent-level telemetry provide an audit trail lacking in most end-to-end neural systems. Preliminary operator studies (not shown) indicate that these artifacts shorten root-cause analysis time by ∼35% compared with baseline ML dashboards.
• **Industrial-scale scalability.** Near-linear scaling on >2,000 GPUs, combined with 150 k objects s^**−**1^ SDA throughput, demonstrates readiness for leadership-class supercomputers. Early edge tests with 8-GPU nodes suggest that pruning the agent society to the most relevant subset retains ≥90% accuracy, hinting at deployability in resource-constrained settings.

**TABLE 6:** Evaluation metrics for Space-Domain Awareness and CFD experiments.

Domain	Primary	Secondary
SDA	Precision, recall, F1; alert latency	Graphs s^**−**1^; GPU utilization
CFD	Final BL-CP (%); mesh-failure rate (%)	Cell count; compute hours

**TABLE 7:** Space-domain awareness performance metrics on held-out three-month set.

Configuration	Precision	Recall	F1-score	Mean alert latency (s)
Full H-I system	0.98	0.98	0.98	2.3
w/o JEPA	0.92	0.93	0.92	3.1
w/o symbolic constraints	0.95	0.94	0.94	2.8
w/o RL planning	0.90	0.91	0.90	4.5

### 4.2 Limitations and open challenges

1. **Data realism.** SDA performance still depends on simulated maneuver catalogs; low-Δv events (<5 cm s^**−**1^) remain a weak spot. Incorporating real-world maneuver logs and higher-fidelity force models is a priority.
2. **Compute overhead.** Although scalable, absolute resource use is high (e.g., 128 GPUs for CFD optimization). Model-compression and iterative-sampling schemes are needed for organizations without leadership-class allocations.
3. **Constraint tuning.** Symbolic rules reduce CFD failures by 86 pp, yet overly strict settings can limit exploration. An adaptive constraint-tuning loop—analogous to temperature schedules in Bayesian optimization—could dynamically relax or tighten rules based on task progress.
4. **Latent interpretability.** JEPA embeddings drive much of the success, but their internal dimensions are still opaque. Visualization probes or concept-activation tests could make latent factors human-readable.
5. **Formal safety proofs.** While constraints catch many invalid states, end-to-end formal verification of multi-agent interactions is still pending.

### 4.3 Future work

• **Adaptive constraint learning.** Coupling symbolic rules with meta-learning could yield task-specific constraints that evolve as data distributions shift.
• **Rich explainability tools.** We plan to generate natural-language rationales and interactive heat-maps that trace causal chains through the agent society—further closing the human-AI trust gap.
• **Edge-optimized deployment.** Lightweight agents and model-distillation pipelines will target 8- to 32-GPU clusters, enabling on-premise industrial use cases.
• **Transfer learning across domains.** Early experiments suggest that CFD mesh-quality embeddings seed faster convergence when fine-tuned on finite-element structural meshes; systematic studies are underway.
• **Hierarchical world models.** Integrating Bayesian or ensemble world models at the planning tier could provide calibrated uncertainty, improving risk-aware decision making.
• **Human-in-the-loop reinforcement.** Active-learning workflows in which monitoring operators label edge cases or override agent decisions, can both enhance safety and reduce labeling cost.

By fusing symbolic precision with data-driven adaptability, the proposed hybrid-intelligence system delivers interpretable, high-performance automation in domains that have historically resisted reliable AI. The demonstrated gains in SDA and CFD, coupled with strong scalability, suggest that such architectures provide a robust foundation for next-generation industrial and defense systems where explainability and trust are as critical as raw accuracy.

## 5 Conclusion

This work introduced a **hybrid-intelligence (H-I) architecture** that blends symbolic reasoning with modern machine-learning, drawing conceptual inspiration from **Global Workspace Theory**. Validated on two demanding domains—space-domain awareness and autonomous CFD mesh generation—the system:

• achieved **F1 = 0.98** in maneuver detection for ∼15,000 space objects,
• raised **boundary-layer capture propagation** to **98%** while cutting mesh-failure rates to **2%**, and
• scaled training pipelines **near-linearly to >2,000 GPUs** on leadership-class supercomputers.

These results confirm that the four mission-critical requirements identified in Section 1—**reliability, explainability, adaptability, and scalability**—can be satisfied simultaneously when symbolic constraints, context-sharing JEPA embeddings, and RL-based planning are engineered to act in concert.

Beyond SDA and CFD, the modular, agent-society design and blackboard transparency provide a transferable blueprint for high-stakes applications such as autonomous energy management, industrial-facility operations, and fusion-plant control. Ongoing work will focus on adaptive constraint tuning, edge-optimized agent distillation, deeper latent-space interpretability, and formal verification of multi-agent safety. Taken together, these directions aim to turn reliable hybrid intelligence from a promising prototype into a routine ingredient of next-generation industrial and defense systems.

## Data Availability

The raw data supporting the conclusions of this article will be made available by the authors, without undue reservation.
